# Comparison between ozone column depths and methane lifetimes computed by one- and three-dimensional models at different atmospheric O_2_ levels

**DOI:** 10.1098/rsos.230056

**Published:** 2023-05-03

**Authors:** A. Ji, J. F. Kasting, G. J. Cooke, D. R. Marsh, K. Tsigaridis

**Affiliations:** ^1^ Department of Geosciences, Penn State University, University Park, PA 16802, USA; ^2^ School of Physics and Astronomy, University of Leeds, Leeds LS2 9JT, UK; ^3^ National Center for Atmospheric Research, Boulder, CO 80301, USA; ^4^ Center for Climate Systems Research, Columbia University, New York, NY 10025, USA; ^5^ NASA Goddard Institute for Space Studies, 2880 Broadway, New York, NY 10025, USA

**Keywords:** atmospheric chemistry, ozone layer, methane lifetime, proterozoic atmosphere, one-dimensional model, three-dimensional model

## Abstract

Recently, Cooke *et al*. (Cooke *et al*. 2022 *R. Soc. Open Sci.* **9**, 211165. (doi:10.1098/rsos.211165)) used a three-dimensional coupled chemistry-climate model (WACCM6) to calculate ozone column depths at varied atmospheric O_2_ levels. They argued that previous one-dimensional (1-D) photochemical model studies, e.g. Segura *et al*. (Segura *et al*. 2003 *Astrobiology* **3**, 689–708. (doi:10.1089/153110703322736024)), may have overestimated the ozone column depth at low pO_2_, and hence also overestimated the lifetime of methane. We have compared new simulations from an updated version of the Segura *et al*. model with those from WACCM6, together with some results from a second three-dimensional model. The discrepancy in ozone column depths is probably due to multiple interacting parameters, including H_2_O in the upper troposphere, lower boundary conditions, vertical and meridional transport rates, and different chemical mechanisms, especially the treatment of O_2_ photolysis in the Schumann–Runge (SR) bands (175–205 nm). The discrepancy in tropospheric OH concentrations and methane lifetime between WACCM6 and the 1-D model at low pO_2_ is reduced when absorption from CO_2_ and H_2_O in this wavelength region is included in WACCM6. Including scattering in the SR bands may further reduce this difference. Resolving these issues can be accomplished by developing an accurate parametrization for O_2_ photolysis in the SR bands and then repeating these calculations in the various models.

## 1. Introduction

**Figure 1.**
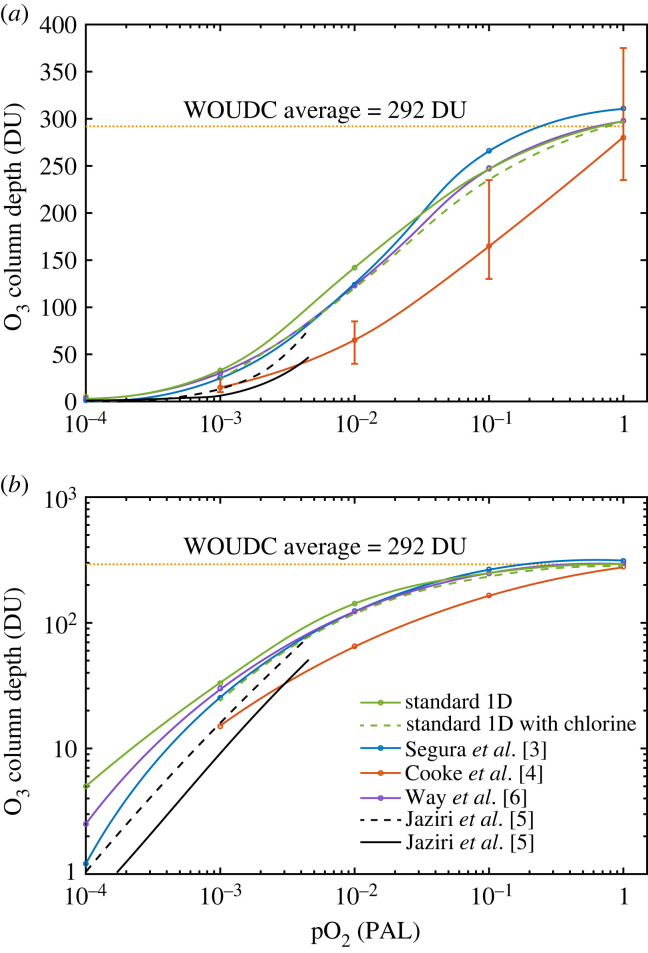
O_3_ column depths at varied pO_2_ levels based on calculations by different groups. Our standard 1-D model is shown by the solid green curve in (*a*) a linear scale and (*b*) a log scale. The standard 1-D model with chlorine is shown by the dashed green curve. ‘WOUDC' stands for ‘World Ozone and UV Radiation Data Center'. Note that we only did calculations at 1 PAL, 0.1 PAL, 0.01 PAL and 0.001 PAL, so the curves do not imply continuous results. In (*a*), the orange curve shows the mean value from WACCM6, with maximum and minimum values (at different longitudes) indicated by the vertical bars. Jaziri *et al*. [5] calculated the ozone column depths for very low pO_2_ (less than or equal to 0.005 PAL) which are also shown here in black curves (dashed black curve for their 1-D calculations at 40° solar zenith angle and solid black curve for their 3-D calculations).

The calculation of ozone column depth as a function of atmospheric O_2_ concentration has been performed multiple times over the past 40 years using one-dimensional (1-D) photochemical models. Several of these calculations [1–3] have been done by the Kasting group. We refer to the current version of that model as the ‘standard' 1-D model (see §2). Recently, Cooke *et al*. [4], henceforth C22, performed a similar calculation using a three-dimensional (3-D) coupled chemistry-climate model (WACCM6). They found substantially lower ozone column depths at lower pO_2_ levels. For example, at 0.01 times the present atmospheric level (PAL) of O_2_, Segura *et al*. [3] calculated 124 Dobson units (DU), whereas C22 [4] calculated a mean column depth of just 66 DU—almost a factor of two lower. This comparison is shown in figure 1. C22 [4] argued, correctly, that 3-D models should be able to do a better job on this problem than 1-D models because ozone column depths are affected by atmospheric transport, e.g. the Brewer–Dobson circulation in the stratosphere that blows ozone from the tropics towards the poles, as well as vertical transport that carries ozone downward from the stratosphere to the troposphere. Meridional transport is absent in 1-D models, and vertical transport is fixed, so model geometry almost certainly contributes to the observed discrepancies in ozone column depth. The 3-D model also has a variable tropopause height (higher at the equator, lower towards the poles), which is difficult to simulate in one dimension.

To complicate matters, an independent 3-D investigation of this problem by Way *et al*. [6] using the ROCKE-3D model produced results similar to the previous 1-D calculations (see C22 [4] fig. 6 and our figure 1), for example, 123 DU at 0.01 PAL O_2_. Another study published in late 2022 by Jaziri *et al*. [5] shows both 1-D and 3-D calculations on the ozone column depths at low pO_2_ (less than or equal to 0.005 PAL) which are much closer to WACCM6 (see black curves in figure 1). A preliminary intercomparison study between the 1-D, WACCM6 3D and ROCKE-3D models suggests that multiple factors contribute to the differences in the modelled ozone column depths. The parametrization of O_2_ photolysis within the Schumann–Runge (SR) bands (175–205 nm) plays a significant role in causing this O_3_ discrepancy, but more detailed work needs to be done to quantify its effect and the effects of other possible factors.

C22 [4] also argued that because of the lower ozone column depths at low pO_2_, the lifetime of CH_4_ should be shorter than previously estimated. That is because less ozone means that more solar near-UV radiation would reach the troposphere, creating more OH to destroy CH_4_ through the following two processes: (i) radiation below approximately 230 nm can directly dissociate gaseous H_2_O, creating OH radicals that then react with CH_4_; (ii) wavelengths between 200 and 310 nm can photolyse tropospheric ozone, creating O(^1^D) radicals that generate more OH by reacting with H_2_O: O(^1^D) + H_2_O→2 OH. As a result, C22 [4] concluded that previous suggestions that methane may have played a significant role in warming the Proterozoic climate are probably incorrect. We demonstrate here that differences in the parametrization of O_2_ photolysis together with whether to consider the absorption by H_2_O and CO_2_ at SR wavelengths account for much of the difference in the methane lifetime, so ruling out methane as an important Proterozoic greenhouse gas would be premature at this time.

## 2. Brief model descriptions

### 2.1. One-dimensional photochemical model

**Figure 2.**
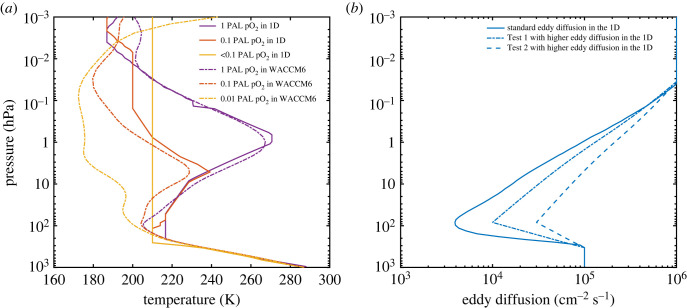
(*a*) Temperature profiles used in the 1-D model and WACCM6 3-D model; different colours represent different pO_2_; solid curves represent 1-D temperature profiles and dashed-dotted curves represent WACCM6 temperature profiles. (*b*) Eddy diffusion profile used in the 1-D model and higher eddy diffusion tests.

The current (Kasting group) 1-D photochemical model is taken directly from Liu *et al*. [7]. It extends from the ground up to 100 km altitude in 1 km increments. This model uses a fully implicit, reverse Euler method to solve the coupled continuity and flux equations, along with a two-stream multiple scattering algorithm from Toon *et al*. [8] to calculate the radiative transfer of incident sunlight for the calculation of photolysis rates only. Three temperature profiles are specified for 1 PAL, 0.1 PAL and pO_2_ of 0.01 PAL and below (figure 2*a*). The modern temperature profile is obtained from US Standard Atmosphere, 1976. These profiles are similar to those calculated by Segura *et al*. [3] using a radiative-convective model. For pO_2_ < 0.1 PAL, we kept the same tropospheric temperature profile and assumed that the stratosphere was isothermal. The model includes standard atmospheric chemistry for C, H, O, N, S species, and we refer to it here as the ‘standard' 1-D model. The main code includes 30 long-lived species, 28 short-lived species and 222 chemical reactions. Some calculations were also performed with chlorine chemistry included, based on the reaction scheme in Stanton *et al*. [9]. Heterogeneous chemistry is not included in the chemical scheme. The treatment of lower boundary conditions for biogenic trace gases (CH_4_, N_2_O, CO, CH_3_Cl and H_2_) is the same as in Liu *et al*. [7] and Segura *et al*. [3]. These trace gases are fixed at observed values for the modern atmosphere simulations. The model returns the surface flux needed to sustain the present values, and we hold these surface fluxes constant and allow the mixing ratios of the various trace gases to float at lower pO_2_ (see electronic supplementary material for details). This low pO_2_ cannot be lower than 10^−4^ PAL in the 1-D model as we design for a well-mixed O_2_ atmosphere. Two important updates have been made, though: (i) eight-point Gaussian integration over the sunlit hemisphere is used in the current 1-D model to more accurately calculate globally averaged photolysis rates (see electronic supplementary material); (ii) new H_2_O cross sections extrapolated to 233 nm by Ranjan *et al*. [10] are included in the current model. Most of the model intercomparisons in this paper are between the updated 1-D model (the standard run) and the WACCM6 3-D model.

### 2.2. WACCM6 model description

The Whole Atmosphere Community Climate Model version 6 (WACCM6) is a configuration of the Community Earth System Model version 2 (CESM2). WACCM6 is a 3-D model which couples together land, land-ice, ocean, sea-ice and atmosphere sub-models [11]. In C22 [4], the simulations used modified configurations of the standard pre-industrial control (the so-called BWma1850 ‘compset'). The atmospheric model uses the finite volume dynamical core [12] with horizontal resolution of 1.875° in latitude by 2.5° in longitude. There were 70 levels in the vertical using a hybrid-sigma coordinate system from the surface to a pressure of 6 × 10^−6^ hPa (approx. 140 km) [11]. At this resolution, the quasi-biennial oscillation (QBO) is produced via a specified dynamical forcing.

The coupled chemistry used in WACCM6 is based on the Model for Ozone and Related chemical Tracers (MOZART; [13–16]). This particular WACCM6 set-up includes 98 chemical species, 208 chemical reactions and 90 photolysis reactions. Twenty-two long-lived species are calculated explicitly; the other 75 species are calculated using a fully implicit Euler backward method with Newton–Raphson iteration [16]. N_2_ is considered to be invariant. For O_3_, Chapman chemistry is included, as well as the HO_x_, NO_x_, BrO_x_ and ClO_x_ species which are involved in catalytic cycles that destroy O_3_. Each constituent is solved individually rather than using a family approach, as in the ROCKE-3D model described below. The atmospheric time step is 30 min. Apart from O_2_, lower boundary conditions were kept the same as in the 1-PAL O_2_ run, whether that was through a fixed flux or a fixed mixing ratio. This means that fixed volume mixing ratios were generally assumed for CH_4_ (8.08 × 10^−7^), N_2_O (2.73 × 10^−7^), CH_3_Cl (4.57 × 10^−10^) and CH_3_Br (5.30 × 10^−12^). Two alternative calculations used fixed fluxes for CH_4_. The standard upper boundary conditions were scaled in order to enable consistent calculations in the upper atmosphere. See C22 [4] for more details. Three new simulations (not described in C22 [4]) were done with a lower amount of methyl chloride, CH_3_Cl (see §5.2), and new simulations at 0.1, 0.01 and 0.001 PAL included the lower boundary conditions from the Kasting 1-D model with SR-band absorption for CO_2_ and H_2_O with updated cross sections.

Reaction rates for WACCM6 were updated to include the Burkholder *et al*. [17] recommendations [11]. Photolysis rates are calculated via a lookup table from the tropospheric ultraviolet and visible (TUV) radiation model [15]. The O_2_ photolysis rate (JO_2_) below 200 nm is calculated for Lyman-α, Schumann–Runge continuum (SRC), and Schumann–Runge bands (SRB), using parametrizations from Chabrillat & Kockarts [18], Brasseur & Solomon [19] and Koppers & Murtagh [20], respectively. JNO is parametrized in the SRB using Minschwaner & Siskind [21]. Scattering is included longward of 200 nm, but not included for O_2_ in the SRB. Additionally, scattering takes place for solar zenith angles beyond 90°. The Koppers & Murtagh [20] scheme is known to be more accurate than the Allen & Frederick [22] band model on which the Kasting group parametrization is based (but see more below on how that parametrization is actually implemented).

WACCM6 simulations have been shown to reproduce the quasi-biennial oscillation (at a higher atmospheric resolution than simulated here), stratospheric sudden warmings, the evolution of Southern Hemisphere springtime ozone depletion over the twentieth century, the water vapour tape recorder in the tropics, the Brewer–Dobson circulation and the observed ozone distribution with latitude [11]. Furthermore, the model has been used to reproduce the evolution of the ozone layer, including the formation of the ozone hole and its recovery [23,24], with observations within the variability limits predicted from the simulations, and larger biases occurring in free-running simulations when compared with specified dynamics simulations [11]. Additionally, WACCM6 is able to reproduce the observed historical evolution of global mean surface temperature anomalies.

### 2.3. ROCKE-3D model description

The Resolving Orbital and Climate Keys of Earth and Extraterrestrial Environments with Dynamics (ROCKE-3D) version 1.0 general circulation model (GCM) was described in Way *et al*. [6], and only a brief description will be provided here. ROCKE-3D is the generalized version of GISS ModelE version 2.0 [25], used in the Coupled Model Intercomparison Project phase 5 (CMIP5), for application to non-modern Earth terrestrial planets. It has been extended to use the Suite Of Community Radiative Transfer codes based on Edwards & Slingo (SOCRATES; [26,27]), among other updates [6]. For the simulations used here, the default model radiative transfer code was used for performance, since the atmosphere was largely similar to that of modern Earth.

The resolution of the model was 2° in latitude by 2.5° in longitude, with a vertical sigma layer coordinate system from the surface to the stratopause, at about 65 km altitude. The time step of the model is 30 min. In the low-O_2_ simulations performed, the atmospheric composition represented that of the pre-industrial atmosphere in all respects except that of lower O_2_ amounts. O_2_ changes were not allowed to affect radiative transfer in climate calculations, i.e. all simulations used present-day O_2_ levels, contrary to O_3_ changes that were taken into account in the radiation calculations. On the other hand, for the photolysis rate calculations, both O_2_ and O_3_ changes were included.

The chemical scheme used is a modified version of the Carbon Bond Mechanism version 4 (CBM4; [28]) as described by Shindell *et al*. [29,30]. The chemical scheme includes an explicit parametrization of O_x_, NO_x_, HO_x_ and CH_4_ reactions, and a heavily parametrized scheme for organic compounds with two or more carbon atoms. In addition, the stratospheric chemistry of chlorine is also included [31], as it is necessary for the simulation of the ozone hole in the late twentieth century. No tropospheric chemistry of halogens is included in the model. Aerosols (primary and secondary organic carbon, black carbon, sulfate, ammonium, nitrate) are included using a bulk scheme (only aerosol mass is simulated), while sea salt and dust are described using a sectional model (Schmidt *et al*. [25] and references therein). There are two wavebands that overlap with the SR bands in the photolysis scheme, centred at 187 and 192 nm. The photolysis scheme includes Rayleigh scattering but does not include direct photolysis of CO_2_ and H_2_O or absorption by these gases (CE Harman, Jr 2023, personal communication).

In simulations with prescribed atmospheric composition, long-lived greenhouse gases (CH_4_, N_2_O, CO_2_, CFCs) are prescribed at the surface using an annually varying (for transient simulations only) global surface mean value, and using a prescribed latitudinal and vertical profile. When chemistry is included in the simulations, which results in atmospheric composition being calculated prognostically, this approach is only used for CO_2_; for the other greenhouse gases (GHGs) that participate in chemistry, only the surface amounts are prescribed, while the values in the vertical are free to evolve as chemistry and dynamics dictate. An important detail is what happens at the surface: at every time step, chemistry and dynamics are allowed to modify surface values, but then these are reset to the prescribed ones. The difference between the calculated and prescribed values is saved and it can be used as a source estimate of those gases. No different treatment was introduced in this approach when the O_2_ levels were changing across simulations.

## 3. Comparisons of ozone column depth as a function of pO_2_

Before discussing the details about how the different models calculate the ozone column depth at varied pO_2_, we first explain some of the parameters that may contribute to the discrepancies in the results.

We compare the temperature profiles used in the 1-D model with the globally averaged temperatures from the WACCM6 model in figure 2*a*. The atmospheric temperature at a particular pressure sets the number density of molecules and influences the rate of many chemical reactions. For the 1-D model, we used three different temperature profiles for 1 PAL O_2_, 0.1 PAL O_2_ and pO_2_ < 0.1 PAL, respectively; detailed descriptions are in §2.1. For the WACCM6 model, we show globally averaged and time-averaged temperature profiles at 1 PAL O_2_ (purple dashed-dotted curve), 0.1 PAL O_2_ (red dashed-dotted curve) and 0.01 PAL O_2_ (yellow dashed-dotted curve). These global mean temperatures for the troposphere in both models are similar, whereas the stratospheric temperatures are quite different (the WACCM6 stratosphere is much colder at low pO_2_). When we substitute the WACCM6 global mean temperature profiles for those in the 1-D model at 1% and 0.1% PAL O_2_, we calculate even higher ozone column depths than in our standard runs (table 1), which makes sense because the rate of formation of ozone and the rates of many ozone-destroying reactions are temperature-dependent. Therefore, while temperature differences are important for ozone column comparisons, they are unlikely to explain all the differences between the standard 1-D model and WACCM6. Because temperature changes with time and space in 3-D models, more work is needed to understand the role of temperature in affecting the mean ozone column depth.

**Table 1.** Ozone column depth (unit: DU) as a function of pO_2_ from different calculations.

no.	pO_2_ (PAL)	1	0.1	0.01	0.001	0.0001
1	standard run^a^	298	248	142	33	5
2	standard run with chlorine species^a^	296	236	120	24	
3	Segura *et al*. [3]^a^	311	266	124	25.3	1.2
4	Cooke *et al*. [4]^b^	279	169	66	18	
5	Way *et al*. [6]^b^	298	247	123	30	2.5
6	Jaziri *et al*. [5] 1-D				18	0.8
7	Jaziri *et al*. [5]^b^				8	0.4
**sensitivity tests in the 1-D model**						
8	a single solar zenith angle (48.2°)^a^	268	224	127	24.5	2.8
9	high H_2_O at the cold trap^a^	287	236	128	24	3
10	fixed CH_3_Cl mixing ratio (0.5 ppb)^a^	296	239	113	5	
11	fixed CH_3_Cl mixing ratio (0.5 ppb) without scattering and H_2_O and CO_2_ absorption from 175 to 250 nm^a^	306	263	13	code crashed	
12	fixed CH_3_Cl surface flux (2.8 × 10^8^ cm^−2^s^−1^) without scattering and H_2_O and CO_2_ absorption from 175 to 250 nm^a^	306	260	155	26	3
13	fixed CH_3_Cl surface flux (2.8 × 10^8^ cm^−2^s^−1^) without scattering and H_2_O and CO_2_ absorption from 175 to 205 nm^a^	298	273	172	29	
14	standard 1-D run but with WACCM6 temperature profiles for 0.01 PAL and 0.001 PAL^a^			153	40	
15	standard 1-D run with high eddy diffusion coefficients	339	273	142	32	
16	standard 1-D run with constant NO production from the lightning	296	259	132	26	
**sensitivity tests in the WACCM6 3-D model**						
17	standard WACCM6 run with 10 times lower CH_3_Cl/CH_3_Br mixing ratio at the surface^b^		180	81		
18	standard WACCM6 run with a billion times lower CH_3_Cl/CH_3_Br mixing ratio at the surface^b^			85		
19	lower boundary conditions from 1-D model and SRB absorption for H_2_O and CO_2_ included^b^		156	55	13	

^a^These calculations were done with the 1-D model described in this paper. (The standard run in the 1-D model is with eight Gauss points, new H_2_O cross section and low-H_2_O profiles.)

^b^These calculations were done with 3-D models.

Another important parameter in the 1-D model is the eddy diffusion coefficient, K, which is derived from empirical studies [32–34]. See the solid blue curve in figure 2*b*. Although theory cannot predict the exact magnitude of K, it provides some guidance on how K might change at lower pO_2_. A decrease in pO_2_ causes a decrease in stratospheric ozone and a corresponding decrease in stratospheric temperature. By reducing or eliminating the stratospheric temperature inversion, this could plausibly increase vertical transport in the lower stratosphere. To determine whether this would affect the ozone column depth in the 1-D model, we increased K in this region (see dashed and dashed-dotted curve in figure 2*b*) and reran the 1-D model. Surprisingly (to us), the ozone column depth actually increased at the higher O_2_ levels (1 and 0.1 PAL) (table 1). That is because the increase in ozone in the troposphere outweighed the decrease in ozone in the stratosphere. At lower O_2_ levels, though, the change in eddy diffusion coefficients had almost no effect on ozone column depth, because the ozone layer had already moved down to the lower stratosphere/upper troposphere. Thus, a change in vertical transport rates seems unlikely to explain the difference between column depths calculated by WACCM6 and by the 1-D model at low pO_2_. Nonetheless, 3-D models calculate vertical transport quite differently, so we cannot rule out this being a factor in the discrepancies between those models and our 1-D model.

### 3.1. Modern O_2_ level

Let us look more closely at the differences in predicted ozone amounts. At 1 PAL O_2_, all four models predict roughly the same column depth, approximately 300 DU. (‘PAL' means ‘times the present atmospheric level'. ‘DU' stands for ‘Dobson units'; 1000 DU = 1 atm cm.) Slight differences are observed (table 1): Segura *et al*. [3] predicted 311 DU; C22 [4] predicted 279 DU; Way *et al*. [6] and our new eight-Gauss-point, 1-D model both predict 298 DU. The standard 1-D result drops to 296 DU when chlorine chemistry is included. The eight-Gauss-point model is a revised version of the Segura *et al*. [3] model and is described further below and more in the electronic supplementary material. It is represented by the green curves in figure 1. For comparison, the World Ozone and UV Radiation Data Center (WOUDC) reported a global average ozone column depth of approximately 292 DU for 1980 [35], but it should be noted that the C22 [4] calculation is for the pre-industrial atmosphere, not the modern atmosphere. The reported WOUDC value decreased by a little over 4% between 1980 and 1994 because of depletion by chlorine compounds and has since recovered.

Before trying to account for the differences between the 1-D and 3-D calculations, we should start by explaining the differences in the older and newer 1-D calculations from the Kasting group. The Segura *et al*. [3] model assumed a diurnal averaging factor of 0.5, i.e. the photolysis rates were multiplied by this fraction. The solar zenith angle was then adjusted to 45° to give approximately the ‘right' ozone column depth for the modern atmosphere. The modern globally averaged ozone column depth was taken from McClatchey *et al*. [36], who listed it as 320 DU. Segura *et al*. calculated 311 DU, so they were still a little on the low side, despite their relatively low solar zenith angle. The Segura *et al*. methodology overestimated the globally averaged solar UV flux by approximately 40% (= cos 45°/cos 60°). Adopting a solar zenith angle of 60° would give the right solar UV flux but would cause the model to severely underestimate the globally averaged ozone column depth.

One strategy for dealing with this issue in a 1-D model is to use the insolation-averaged daytime solar zenith angle of 48.2° suggested by Cronin [37]. Combining this with a diurnal averaging factor of 0.375, or 3/8, gives the right globally averaged solar flux, along with a reasonable ozone column depth of 268 DU. We used that strategy in a recent paper by Liu *et al*. [7]. That number is consistent with the revised lower estimate of ozone column depth based on observations. Here, we go a step further and use an eight-point Gaussian integration over the sunlit hemisphere (see electronic supplementary material). This procedure places some weight on calculations at very low solar zenith angles (Sun high in the sky) that allow more photons to reach the lower stratosphere and, thus, increase the column-integrated O_2_ photolysis rate. This creates more ozone compared with the 48.2° model, bringing it closer to the WOUDC value.

**Figure 3.**
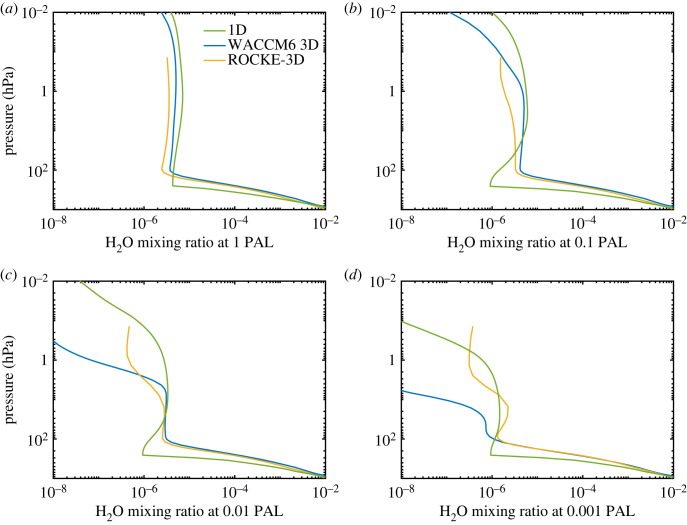
H_2_O mixing ratio at different values of pO_2_ in the 1-D (green curve), WACCM6 3D (blue curve) and ROCKE-3D (yellow curve) models.

Our present 1-D model and the one used by Segura *et al*. [3] differ in at least two more important ways:

1) The Segura *et al*. model included chlorine chemistry, whereas our ‘standard' 1-D model does not. But, as noted earlier, we added chlorine chemistry back into the model for some of the calculations done later during this study. The assumed source of chlorine in the Segura *et al*. model was 0.5 ppbv of methyl chloride, CH_3_Cl, near the surface, supported by a flux of 7.29 Tg(CH_3_Cl) yr^−1^. (This flux drops to 3.91 Tg(CH_3_Cl) yr^−1^ in the standard model with chlorine discussed here, because of the change in how photolysis rates are calculated). (Recall that the Segura *et al*. model had too many solar UV photons.) The methyl chloride *flux* was held constant in calculations at lower O_2_ levels. We return to the question of lower boundary conditions in §5.1 because the predicted concentrations of other biogenic species (e.g. CH_4_ and N_2_O) also depend critically on this parameter.
2) Our new model assumes less H_2_O in the upper troposphere than did the model of Segura *et al*. [3]. Segura *et al*. used a tropospheric relative humidity (RH) profile from Manabe & Wetherald [38]. That model yields approximately 6 ppmv at the tropopause cold trap, considerably higher than the values seen in the two 3-D models with which we compare. (The tropopause is set at 10 km, or approximately 300 mbar, in the 1-D model.) So, in the new model we modified the RH profile to produce approximately 4 ppmv H_2_O at the tropopause. By comparison, the ROCKE-3D model has about 2 ppmv H_2_O in the lower stratosphere, and the WACCM6 3D model has approximately 3 ppmv H_2_O (figure 3*a*). The 3-D models have a cold, high equatorial tropopause that effectively vacuums water vapour out of the stratosphere. The H_2_O concentration increases with altitude in the stratosphere in all three models as a result of methane oxidation. By comparing the ozone column depth in the high-H_2_O version of the 1-D model (287 DU, case 9 in table 1) with that in our standard 1-D model (298 DU, case 1 in table 1), it can be seen that the high-H_2_O model results in roughly a 3% (21 DU) decrease in ozone column depth at 1 PAL O_2_. More details are provided in §1.1.2 in electronic supplementary material.

### 3.2. Lower O_2_ levels

**Figure 4.**
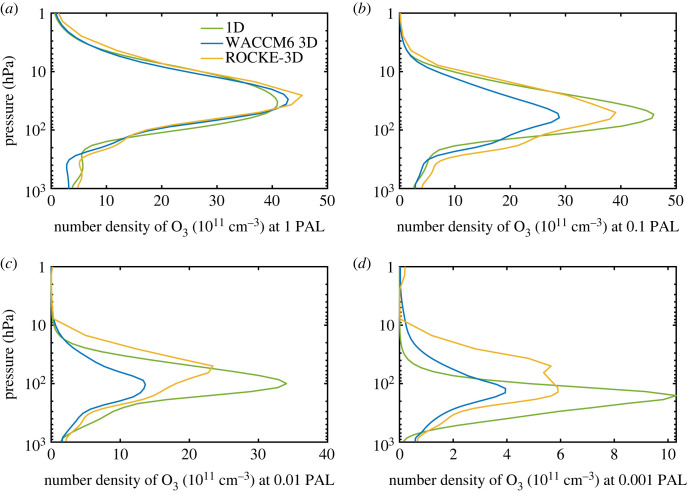
Comparison of ozone number density at different values of pO_2_ in the 1-D (green curve), WACCM6 3D (blue curve) and ROCKE-3D (yellow curve) models.

While the three models agree fairly well on the ozone column depth of the present atmosphere, the predictions of the WACCM6 model diverges from the other two models at low pO_2_. For example, the discrepancy between WACCM6 and the standard 1-D model is already a factor of 1.5 at 0.1 PAL O_2_ and rises to 2.2 below that O_2_ level (figure 1 and table 1). The column depth in the 1-D model is consistently higher than WACCM6 at low pO_2_. ROCKE-3D remains close to the 1-D model down to 10^−3^ PAL but predicts only half as much ozone below that. (WACCM6 does not have a calculation at 10^−4^ PAL O_2_.) The Jaziri *et al*. [5] 1-D model column densities agree with WACCM6 at 10^−3^ PAL, while the 3-D model columns are about a factor of 2 less. At 10^−4^ PAL both of these simulations are less than the 1-D model values. Note that when chlorine chemistry is included, the new 1-D model predicts ozone column depths that are close to those calculated by Segura *et al*. [3]. This is somewhat surprising, given the lower (and more correct) UV fluxes calculated in the newer model. But it may simply show that the calculation is relatively insensitive to the solar UV flux, because O_2_ and H_2_O photolyse at roughly the same wavelengths. To look more closely at where these discrepancies arise, we compare vertical profiles of ozone number density at different pO_2_ levels (figure 4). Ozone is shown on a linear scale to highlight the differences in the stratosphere, where most of the ozone is found. At low pO_2_, the peak number density of ozone in the 1-D model is always larger than that in the two 3-D models, and the WACCM6 model always shows the smallest peak number density. For example, the peak ozone density in the WACCM6 model is approximately$\;1.4\times 10^{12}{\;\mathrm{cm}}^{-3}$ at 0.01 PAL and approximately $\;4\times 10^{11}{\;\mathrm{cm}}^{-3}$ at 0.001 PAL, which is less than half that predicted by the 1-D model for those O_2_ levels (figure 4*c,d*). At low pO_2_, the ozone layer is also more strongly peaked in the 1-D model than in either 3-D model.

Note also that the peak ozone density occurs lower in the atmosphere as the O_2_ concentration decreases. (This is not a new result: it was pointed out 50 years ago by Ratner & Walker [39].) The ozone layer migrates downward as pO_2_ decreases because solar UV radiation capable of dissociating O_2_ penetrates more deeply into the atmosphere. By the time pO_2_ has decreased to 10^−3^ PAL, the peak ozone density in the 1-D model has moved down to approximately 200 mbar (12 km)—a region that is much drier in the 1-D model than in the two 3-D models (figure 3*b–d*). Lower H_2_O in this region implies lower HO_x_ production, and that in turn should imply less destruction of O_3_. The two important reaction sequences are

3.1 $$O_{3}+hv\to O_{2}+O{(}^{1}D),$$

3.2 $$O{(}^{1}D)\;+H_{2}O\to 2\mathrm{OH}\;$$

and

3.3 $$H_{2}O+hv\to \mathrm{OH}+H.$$

Reactions (3.1) and (3.2) dominate OH production in the modern atmosphere, but reaction (3.3) becomes important at low pO_2_ (see §4). We suspect that some of the differences between the 1-D model and WACCM6 may arise from differences in upper tropospheric H_2_O concentrations. Indeed, comparison of Case 1 and Case 9 in table 1 shows that the high-H_2_O version of the 1-D model has 27% less ozone than the standard model at 0.001 PAL O_2_. So, this is a big effect. But HO_x_ production also depends on H_2_O photolysis rates, which are affected by shielding of H_2_O by O_2_ and O_3_. O_2_ photolysis is particularly critical and is calculated differently in each model. We examine these factors more closely in the next section.

Before doing that, though, we should mention the two black curves in figure 1*a*,*b*. These show calculations by Jaziri *et al*. [5], in which they compared ozone column depths calculated with a 3-D model and a 1-D model that share the same photochemical scheme. This paper, which came out too recently to be included in the present intercomparison, was focused on a different problem, namely, the transition from anoxic to oxic conditions, sometimes called the Great Oxidation Event (GOE). Their calculations were performed for O_2_ mixing ratios of 10^−3^ or below (pO_2_ ≤ 0.005 PAL), so they overlap with the other models only at the lowest O_2_ levels studied here. They also included 100 ppmv of CH_4_, omitted halogen and nitrogen chemistry, and did not include gravity waves, which contribute to the forcing of the Brewer–Dobson circulation. Thus, their calculations are not directly comparable to the models discussed here, but inclusion of these processes would probably reduce the ozone column amounts they report. Their calculations show something that may very well be significant: the ozone column depths calculated by their 3-D model were always significantly lower than those found in their 1-D model (we show their 1-D calculation with a solar zenith angle of 40^o^, which is close to the 45^o^ zenith angle used by Segura *et al*. [3]). This suggests that 1-D calculations are missing some fundamental process that tends to reduce ozone levels at low pO_2_, supporting the argument made in C22 [4]. We return to this point in §5.2.

## 4. Photolysis rates of H_2_O and O_2_

### 4.1. Validation of the 1-D model at 1 PAL O_2_

**Figure 5.**
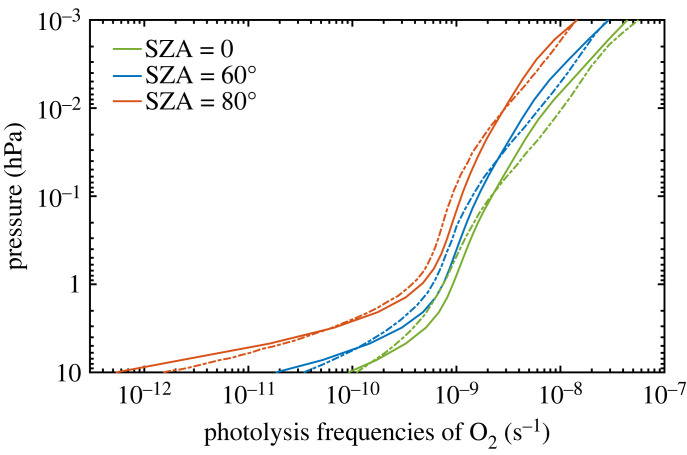
Comparison of photolysis frequencies of O_2_ for the modern atmosphere in the Kasting group 1-D model (solid curves) and from the model of Fernández *et al*. [40] (dashed curves). SZA, solar zenith angle.

Before attempting to compare O_2_ photolysis rates in the three photochemical models, we should first ask: do any of them agree with the most up-to-date calculations of O_2_ photolysis rates? As discussed further below, the difficult part of this calculation is to estimate absorption in the O_2_ Schumann–Runge bands between 175 and 205 nm. An accurate line-by-line calculation of O_2_ photolysis rates in the modern atmosphere was performed by Fernández *et al*. [40] using data from the HITRAN database (https://hitran.org/). This calculation was done for the present O_2_ level using the 1976 US Mid-latitude Standard Atmosphere temperature profile. The same modern temperature profile is used in the 1-D model (figure 2*a*). Photolysis rates of O_2_ from that model and from Fernández *et al*. were computed at 0^o^, 60^o^ and 80^o^ solar zenith angles (figure 5). These calculations were done for the dayside, i.e. they did not include diurnal averaging. Photolysis rates calculated by the two models are in reasonably good agreement at all altitudes except near the very top of the atmosphere, where Fernández *et al*. compute higher rates. (This does not matter much for ozone column depth because most of the ozone is found at lower altitudes.) What look like small differences on the log scale used in figure 5 can actually be quite significant, though. At a solar zenith angle of 0^o^ and an altitude of 40 km, for example, the photolysis rate predicted by our 1-D model is higher than that found by Fernández *et al*. by approximately 25%. This difference occurs near where odd oxygen (O_x_) production peaks and may account for the differences in O_x_ production between the 1-D model and WACCM6 (see next section).

### 4.2. Photolysis of O_2_ in the Schumann–Runge bands

**Figure 6.**
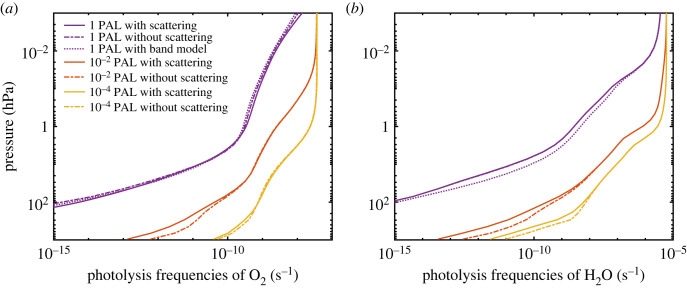
Photolysis frequencies of O_2_ (*a*) and H_2_O (*b*) calculated in the 1-D model with and without scattering at three different O_2_ levels.

As already mentioned, photolysis of O_2_ in the SR bands plays a key role in these calculations. The 1-D model uses an exponential sum formulation for the O_2_ absorption coefficient which allows that model to include multiple scattering. The exponential sums in the 1-D model were derived more than three decades ago by fitting the Allen & Frederick [22] band model at 1 PAL O_2_ (see electronic supplementary material). As a first step towards testing this parametrization at lower pO_2_, we show calculated O_2_ photolysis frequencies versus altitude for three different atmospheric O_2_ levels in figure 6*a*. Calculations were performed using the exponential sum formulation, both with (solid curves) and without (dashed curves) scattering. When scattering is neglected at 1 PAL O_2_, the photolysis rates of O_2_ and H_2_O are almost unaffected, as expected. Note also that the no-scattering calculation agrees well with the original Allen and Frederick band model at 1 PAL O_2_, as it should. Significant differences arise at low-O_2_ levels, however. At 0.01 PAL O_2_, the photolysis rate of O_2_ at the surface is five times higher when scattering is neglected, and the photolysis rate of H_2_O is higher by a factor of 8. The reason is that the effective path length of the incoming photons is significantly increased when multiple scattering is included. So, it is clear that multiple scattering at SRB wavelengths should be included when performing these low-O_2_ calculations. But one should also realize that the exponential sum coefficients calculated at 1 PAL O_2_ may not be accurate at lower pO_2_ because the temperatures and pressures in the region where SR absorption occurs can be quite different. So, even the 1-D parametrization with scattering is less than ideal. We explain how this problem can be corrected in §6.

The WACCM6 3-D model used by C22 [4] parametrizes absorption/photodissociation in the O_2_ SR bands using a method developed by Koppers & Murtagh [20] and Marsh *et al*. [41]. This method does not include scattering within the SR bands. For 1 PAL O_2_ conditions, scattering in the SR bands is not important and the parametrization used in WACCM6 agrees well with the line-by-line calculations. The implementation of this algorithm in the WACCM6 model previously neglected absorption of SR-band radiation by H_2_O and CO_2_. That is a separate issue that will be discussed in the next section. Neglecting scattering is a valid approximation in the present atmosphere because most solar radiation at these wavelengths is absorbed above 60 km, where the air is thin. But at 0.01 PAL O_2_, the relative importance of Rayleigh scattering (most of which is done by N_2_) should be higher by a factor of 100 compared with today because solar UV radiation will penetrate deeper into the atmosphere. Thus, scattering at SRB wavelengths should be included when performing calculations at low pO_2_.

### 4.3. Comparison of photolysis rates in the 1-D and 3-D models

**Figure 7.**
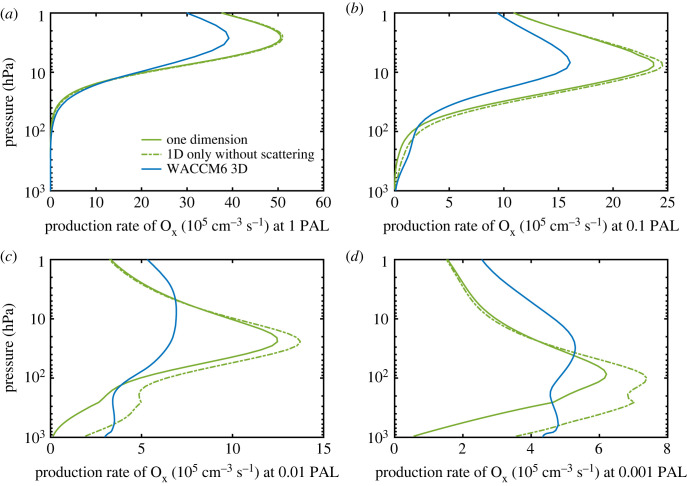
Production rate of odd oxygen (O_x_) at different values of pO_2_ in the standard 1-D model (solid green curve), the standard 1-D model without scattering (dotted-dashed green curve) and the WACCM6 3-D model (globally averaged, solid blue curve).

Comparisons of globally averaged O_2_ and H_2_O photolysis frequencies (in units of s^−1^) calculated by the 1-D model and by WACCM6 are shown in electronic supplementary material, figure S5. Here we show in figure 7 the globally averaged odd-oxygen production rate (in units of cm^−3^ s^−1^) as a function of pressure, which highlights fundamental differences between the 1-D model and the WACCM6 3-D model. The standard 1-D model predicts higher (and more strongly peaked) O_2_ photolysis rates than does WACCM at 0.1 and 0.01 PAL O_2_. At 1 and 0.1 PAL O_2_, the odd oxygen production rate around 3 mbar hPa^−1^ (approx. 40 km) is about 25% higher in the 1-D model than in the 3-D model. At 0.01 and 0.001 PAL O_2_, O_x_ production is almost twice as high in the 1-D model and is much more strongly peaked. As mentioned earlier in §4.1, the difference in O_2_ photolysis rates between the 1-D model and Fernández *et al*. line-by-line calculation is about 25% at approximately 40 km in the modern atmosphere. This may explain, at least partly, the 25% difference for O_2_ photolysis rate between the 1-D model and WACCM6 3-D model at higher O_2_ levels. To say this differently, for the modern atmosphere the O_2_ photolysis rates from WACCM6 agree with Fernández *et al*. [40] better than those computed by the 1-D model, probably because WACCM6 uses a more up-to-date parametrization of the SR bands (see next section). We also show in electronic supplementary material, table S2 that applying WCCM6 UV fluxes, which are lower than that in the SR bands in the 1-D model, leads to an approximately 10% reduction in total column-integrated O_2_ photolysis rate at 0.01 PAL, but still cannot explain the large proportion of the discrepancies in the ozone column depth.

### 4.4. OH concentrations at low-O_2_ levels

**Figure 8.**
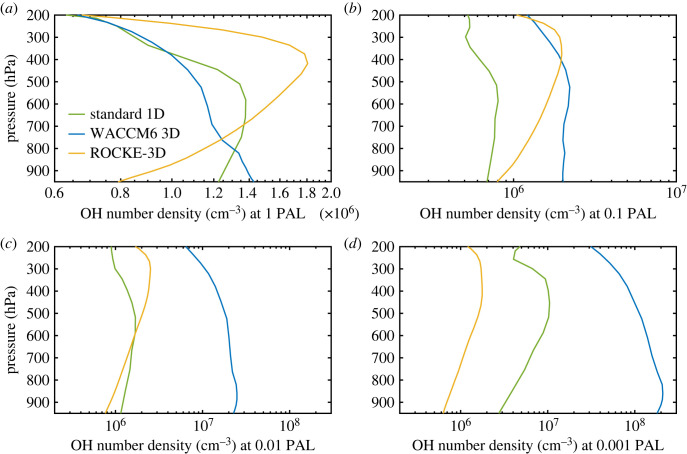
OH number density as a function of pO_2_ in standard 1-D (green curve), WACCM6 3D (blue curve) and ROCKE-3D (yellow curve) models.

Photolysis of H_2_O is particularly important at low pO_2_ because it leads to production of OH, which is the most important oxidant in both the modern and ancient troposphere. As shown in figure 8, tropospheric OH concentrations are very different in the three models at some O_2_ levels. Figure 8*a* shows comparisons at 1 PAL of O_2_. The 1-D model predicts an OH number density, *n*_OH_, of 1.25 × 10^6^ cm^−3^ at the surface. The *n*_OH_ from WACCM6 is about 15% higher; *n*_OH_ from ROCKE-3D is about 30% lower. Peak tropospheric OH densities occur at 600 hPa (approx. 4 km) in the 1-D model (approx. 1.4 × 10^6^ cm^−^^3^), 400 hPa (approx. 7 km) in ROCKE-3D (approx. 1.8 × 10^6^ cm^−^^3^), and at the surface in WACCM6 (approx. 1.45 × 10^6^ cm^−^^3^). As a comparison, the standard reference of annually and globally averaged OH concentration peaks around 1.5 × 10^6^ cm^−^^3^ at 700 hPa in Spivakovsky *et al*. [42] (see their fig. 8). The reason for these discrepancies is probably due to the different treatments of the absorption by H_2_O and CO_2_ within the SR bands, and we cannot rule out other factors that may be related to the spatial inhomogeneity of OH, including the cloud distribution in the 3-D models. The 1-D model atmosphere is cloud-free. Also note that the OH profiles in the WACCM6 3D simulations differ from those in Spivakovky *et al*. [42] because WACCM6 used here assumes a pre-industrial atmosphere without pollutants.

**Figure 9.**
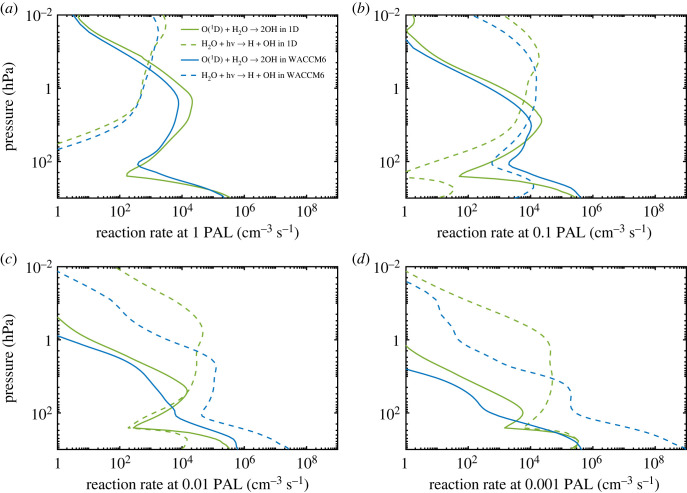
OH production from the reaction of O (^1^D) with H_2_O and from photolysis of H_2_O at different O_2_ levels in the 1-D and WACCM6 3-D models.

At low pO_2_, the discrepancies in OH density are much larger. We focus here on the most extreme case: surface OH densities at 0.001 PAL O_2_. As shown in figure 8*b*, the 1-D model predicts *n*_OH_ = 3 × 10^6^ cm^−3^, ROCKE-3D predicts 2.2 × 10^5^ cm^−3^ (14 times lower than the 1-D model), and WACCM6 predicts 2 × 10^8^ cm^−3^ (almost 70 times higher than the 1-D model). The low surface OH number densities predicted by ROCKE-3D are easily explained, as this model does not include H_2_O photolysis at SR-band wavelengths (reaction (3.3) in §3.2). H_2_O photolysis is a major source of tropospheric OH at low pO_2_ (figure 9). The disagreement between WACCM6 and the 1-D model can be explained in a similar manner. At high pO_2_, tropospheric OH is produced mostly by reaction (3.2) (§3.2), but at low pO_2_, reaction (3.3) becomes important. The standard 1-D model, which was designed for low-O_2_ levels, handles this transition self-consistently. But WACCM6, which was designed for the modern atmosphere, does not. As pointed out in the previous subsection, WACCM6 neglects scattering within the wavelength region of the SR bands, and it does not treat either H_2_O or CO_2_ as major absorbers of solar UV radiation in C22 [4]. (It allows these species to photolyse, but it does not consider their effect on the UV opacity.) The latter assumption is acceptable for the modern atmosphere because CO_2_ is scarce and because radiation at these wavelengths is absorbed high in the atmosphere where H_2_O is even scarcer. At 0.001 PAL O_2_, however, CO_2_ is 50% more abundant than O_2_ (assuming modern CO_2_ levels), and SR-wavelength radiation penetrates to the lowest atmospheric layer where H_2_O is more than 50 times as abundant as O_2_.

**Figure 10.**
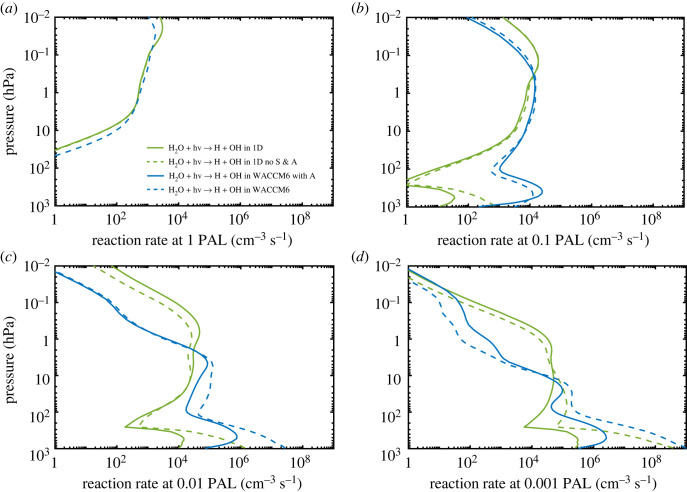
Comparisons of the rate of H_2_O photolysis at different O_2_ levels in the 1-D (green curves) and WACCM6 3-D (blue curves) models. The solid green curve shows the standard run in the 1-D model, and dashed green curve shows the same run without scattering and without treating CO_2_ and H_2_O as major absorbers at SR wavelengths (no S & A). The solid blue curve shows the WACCM6 simulation with absorption by H_2_O and CO_2_, and the dashed blue curve shows the WACCM6 simulation without absorption by H_2_O and CO_2_.

**Figure 11.**
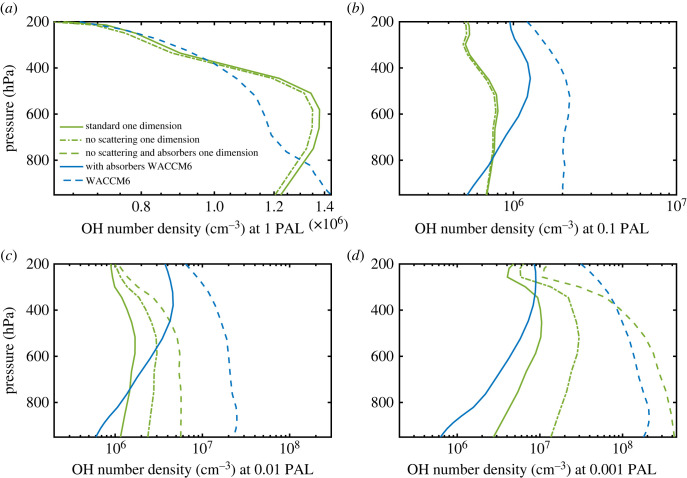
OH number density as a function of pO_2_ in the standard 1-D model (green curves) and the WACCM6 model (blue curves). In the 1-D model, solid green curves represent results from the standard runs, dashed green curves represent results without scattering and absorption by H_2_O and CO_2_ between 175 and 250 nm, and dashed-dotted green curves show the simulation without scattering in this same wavelength region. In the WACCM6 model, solid blue curves represent results with absorption by H_2_O and CO_2_ between 175 and 205 nm, and dashed blue curves represent results without absorption by H_2_O and CO_2_ in this wavelength region.

To test the impact of omitting these factors, we eliminated scattering between 175 and 250 nm in the 1-D model and treated H_2_O and CO_2_ as minor absorbers within this same wavelength region. The results are shown in figure 10. At 0.001 PAL, OH production near the surface increases by more than a factor of 1000 compared with our standard 1-D simulation, yielding almost as much OH production as seen in WACCM6 model (figure 10*d*). This comparison explains why tropospheric OH concentrations are so much higher in WACCM6. These calculations were run for only one time step, so that they started from the same background atmosphere. By running the 1-D model to convergence, we can look more closely at how the OH number density would change when scattering and absorbers are neglected at 0.001 PAL. The result is shown in figure 11. The OH number density near the surface increases by more than a factor of 200. We also tested only the scattering effect at two different wavelength ranges: SR bands (175–205 nm) and Herzberg continuum (205–250 nm) because WACCM6 does have scattering between 200 and 250 nm. We found that OH production at low pO_2_ is mainly affected by the treatment of the SR bands, and whether we include scattering within the Herzberg continuum does not make a measurable difference.

The effect of H_2_O/CO_2_ absorption on OH production and thus its concentration is also noticeable in WACCM6 after adding the absorption. For example, at 0.001 PAL, the new simulation in WACCM6 including H_2_O/CO_2_ absorption predicts OH production near the surface will decrease to almost the same level as in the 1-D model (figure 10*d*). This also leads to a decrease by more than a factor of 200 in OH number density in the new WACCM6 simulation at 0.001 PAL (figure 11*d*). So, this discrepancy can be considered resolved.

## 5. Why predicted ozone column depths and methane lifetimes differ at low-O_2_ levels

We are now in a somewhat better position to understand why the ozone column depths and methane lifetimes predicted by the 1-D and 3-D models differ at low-O_2_ levels. Both issues are at least partly related to tropospheric OH concentrations. We start with the methane lifetime question, as that discrepancy is now fully understood.

### 5.1. Methane lifetimes at low-O_2_ levels

In addition to predicting lower ozone column depths at low pO_2_, C22 also predicted that methane should have a much shorter lifetime than predicted in previous 1-D modelling efforts. C22 [4] suggested that these two phenomena were closely related: the lower ozone column depths allowed more solar UV radiation to penetrate to the troposphere, creating more O(^1^D) from ozone photolysis (reaction (3.1)), and thereby increasing OH concentrations and lowering the lifetime of CH_4_. The two major photochemical loss processes for methane in the modern atmosphere are the reactions

5.1 $${\mathrm{CH}}_{4}+\mathrm{OH}\to {\mathrm{CH}}_{3}+H_{2}O,$$

and

5.2 $${\mathrm{CH}}_{4}+O{(}^{1}D)\;\to {\mathrm{CH}}_{3}+\mathrm{OH}.$$

These reactions remain the dominant loss processes for methane at all O_2_ levels studied here. Methane can also be photolysed directly, but this reaction happens only at very short wavelengths (less than 145 nm) and thus occurs high up in the atmosphere, even at low pO_2_.

Because methane is not produced within the atmosphere, methane lifetimes in all three models can be calculated by dividing the CH_4_ column depth by the CH_4_ input flux at the surface, or by integrating the total loss rate across the whole atmosphere when the mixing ratio is fixed. (‘Surface' methane lifetimes shown in table 2 of C22 [4] are not used for this comparison because such a calculation considers only CH_4_ loss in the lowermost atmospheric layer.) Because methane is well-mixed in the lower atmosphere at all O_2_ levels, the methane column depth can be approximated as the CH_4_ number density in the bottommost layer times the atmospheric scale height (about 7.5 km).

**Figure 12.**
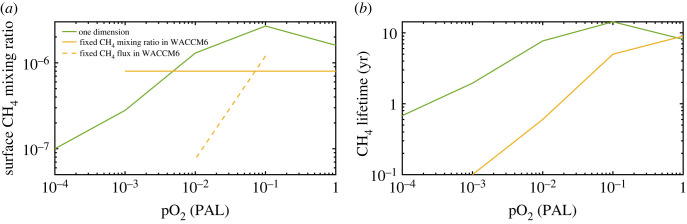
Surface CH_4_ mixing ratio (*a*) and CH_4_ lifetime (*b*) at different pO_2_ levels in the 1-D (solid green curve), WACCM6 3-D (yellow curve) models. The solid yellow curve in (*a*) shows fixed CH_4_ mixing ratio (0.8 ppmv) runs in WACCM6, whereas the dashed yellow curve shows fixed CH_4_ flux (500 Tg yr^−1^) runs. The WACCM6 methane lifetimes in (*b*) were calculated from the fixed mixing ratio runs.

Comparisons of methane concentrations and lifetimes between the 1-D model and WACCM6 model are shown in figure 12. The 1-D model calculations were done by fixing the surface CH_4_ mixing ratio at its (approximate) modern value, 1.6 ppmv, at 1 PAL O_2_. The model then calculates the CH_4_ flux (1.13 × 10^11^ cm^−2^ s^−1^) needed to support it. In geochemists' units, this is equivalent to a flux of 480 Tg(CH_4_) yr^−1^. This yields a CH_4_ lifetime of 8.0 years for the modern atmosphere, which is close to the accepted value in Kirschke *et al*. [43]. At lower O_2_ levels, the CH_4_ flux was held constant, and the model calculated its surface mixing ratio (figure 12*a*). The CH_4_ mixing ratio—and, thus, its lifetime—peaks at 0.1 PAL O_2_. This peak appears to be caused by lightning. In today's atmosphere, lightning is the major non-anthropogenic source of nitrogen oxides in the troposphere via the high-temperature reaction: N_2_ + O_2_ ↔ 2NO. The 1-D model scales the column-integrated rate of NO production to lower O_2_ levels by assuming thermodynamic equilibrium at a ‘freeze-out’ temperature of 3500 K in the expanding, cooling, cylindrical shock wave surrounding the lightning bolt. As O_2_ drops to 0.1 PAL, the NO production rate decreases to just one-third of that for today, based on this parametrization. According to models of smog chemistry, NO reacts with the by-products of methane (or higher hydrocarbon) oxidation to produce O_3_, and this, in turn, leads to more OH through reactions (3.1) and (3.2). Thus, at 0.1 PAL O_2_, less NO leads to less O_3_, less OH and a longer methane lifetime. As O_2_ continues dropping below this level, more OH is produced by reaction (3.3), which leads to a decrease of CH_4_ lifetime. The CH_4_ mixing ratio falls to approximately 0.1 ppmv at 10^−4^ PAL O_2_, and its lifetime decreases to about eight months. The changes in tropospheric O_3_ caused by this mechanism are too small to account for the discrepancies in ozone column depth.

The WACCM6 model described in C22 [4] was operated in two different modes. One set of calculations was done in a similar manner as the 1-D calculations, except that the standard calculation was done for the pre-industrial atmosphere (0.8 ppmv CH_4_). This yielded a surface CH_4_ flux of 5.6 × 10^10^ cm^−2^ s^−1^ (approx. 240 Tg(CH_4_) yr^−1^), which corresponds to a CH_4_ lifetime of 9 yr. Fixed fluxes of 50, 500 (present level), and 5000 Tg(CH_4_) yr^−1^ were then used as the lower boundary condition for CH_4_ in calculations done at 0.1 and 0.01 PAL O_2_. (The 0.1 PAL run was done subsequent to the publication of C22 [4].) Results for the 500 Tg(CH_4_) yr^−1^ run are shown in figure 12*a*. In another set of WACCM6 calculations, the CH_4_ mixing ratio was held fixed at 0.8 ppmv at all O_2_ levels (figure 12*b*). In these simulations, the CH_4_ lifetime dropped to as low at 0.1 yr at 0.001 PAL O_2_. Thus, a very large CH_4_ flux (approx. 80 times the present value) would have been needed to sustain such a mixing ratio if this calculation was correct. But we showed in the previous section that tropospheric OH concentrations at 0.001 PAL may be overestimated by a factor of 200 in the WACCM6 model because of the treatment of O_2_/H_2_O photolysis. So, these calculated lifetimes could be as much as 200 times too low at 0.001 PAL. In addition, the total NO_x_ production from lightning in the C22 [4] does not scale to lower pO_2_; instead, it is almost constant for each O_2_ level (see details in electronic supplementary material). This higher NO compared with the 1-D model at 0.1 PAL could result in more O_3_, and thus more OH. Therefore, shorter CH_4_ lifetime would be expected as C22 [4]. But the lightning effect at much lower pO_2_ (less than 0.1 PAL) on CH_4_ lifetimes is not expected to be solved in this paper. The actual underestimation of the methane lifetime is probably smaller than this because the discrepancies in calculated OH abundances decrease with altitude.

As an aside, another biogenic trace gas, N_2_O, was also treated with fixed mixing ratio (270 ppbv) lower boundary conditions in the WACCM6 model. This also results in high surface fluxes at low pO_2_. At 10^−4^ PAL O_2_, for example, the 1-D model calculates an N_2_O surface mixing ratio of approximately 0.8 ppbv, about 0.3% of the present value. Hence, keeping N_2_O fixed at 300 ppbv in the 1-D model would require a surface flux more than 300 times higher than today. This assumption is unlikely to account for the lower ozone column depths found by WACCM6, however, because at low pO_2_ most N_2_O photolyses back to N_2_ + O rather than reacting with O(^1^D) to produce NO.

### 5.2. Ozone column depths at low pO_2_

Although the reason(s) for the lower ozone column depths at low pO_2_ in the WACCM6 model are not fully resolved, we do understand at least some of the issues. We number these below, for clarity:

1) Lower boundary conditions and chlorine chemistry clearly affect the calculated ozone column depths. We have done several sensitivity analyses on chlorine in both the 1-D and WACCM6 models. The results are listed in table 1 and details can be found in the electronic supplementary material. The 1-D model is extremely sensitive to the lower boundary condition on CH_3_Cl, especially when scattering and absorption by H_2_O/CO_2_ is neglected at SR wavelengths (see cases 10 and 11 in table 1). In case 11, the ozone column depth decreases by a factor of 10 at 0.01 PAL O_2_ when a fixed mixing ratio boundary condition is used for CH_3_Cl. At 0.001 PAL O_2_ ozone basically disappears and the code fails to converge. But the WACCM6 model appears to be much less sensitive to changes in lower boundary conditions (see cases 17–19 in table 1).
2) A second factor that clearly affects ozone column depths is upper tropospheric H_2_O densities. These are higher in WACCM6 than in the 1-D model because WACCM6 has a high tropical tropopause, allowing convection to bring H_2_O into the upper troposphere. Sensitivity experiments described in §§3.1 and 3.2 show that the 1-D model predicts less ozone when upper tropospheric H_2_O levels are increased. This effect is particularly pronounced at low pO_2_, partly explaining why the 3-D model predicts less ozone in these conditions. This may also help explain why the Jaziri *et al*. [5] 3-D model predicts less ozone than their 1-D model. But it does not explain why the same effect is not observed in the ROCKE-3D calculation.
3) The different parametrizations of O_2_ photolysis in the SR bands cause O_x_ production to be lower in WACCM6 than in the 1-D model. This issue can best be resolved by developing a more robust parametrization of absorption at these wavelengths (see §6).
4) Vertical transport is also very different in the two models. In the 1-D model, vertical transport is parametrized as eddy diffusion and is fixed at all pO_2_ levels. By contrast, in WACCM6, as O_2_ is decreased, the reduced levels of O_3_ result in less stratospheric heating. Because the stratospheric polar vortex forms due to latitudinal temperature gradients, when O_3_ is reduced, the average velocity of the polar vortex decreases. This change in wind fields seen in the WACCM6 simulations, including a weakened Brewer–Dobson circulation, is consistent with previous work [44,45] and affects the latitudinal distribution of O_3_ (more details are included in the electronic supplementary material). The other 3-D models also have sophisticated vertical transport schemes, but the fact that they do not all agree shows that this problem is complicated.
5) Other factors that contribute to differences in calculated ozone column depths include: the temperature structure which modulates reactions rates and densities; chemical mechanism differences (e.g. assumed reaction rates, or the inclusion of heterogeneous chemistry on polar stratospheric clouds); and the inclusion of seasons and the diurnal cycle. While we believe these effects are small compared with the four factors discussed just above, future work should quantify their importance.

## 6. Suggestions for future research

As we have shown, a critical part of the photochemical schemes of all three models involves photolysis rates in the O_2_ SR bands. None of the models compared here treats this problem rigorously. The Kasting *et al*. 1-D model may come closest because it includes multiple scattering at these wavelengths, but its limitations were pointed out earlier. A number of improved models of the SR bands have been published during the past 25 years [21,46–48]. These models presumably do an excellent job of parametrizing SR band absorption by O_2_ in the modern atmosphere, but they may still not be optimal for low-O_2_ atmospheres in which scattering is very important at these wavelengths. A widely used tool in calculating atmospheric absorption in the thermal-IR is that of correlated-k coefficients [49,50]. The Fernández *et al*. model, mentioned earlier, or some other line-by-line model, could be used to generate broadband, correlated-k coefficients for O_2_ absorption that would remain valid for atmospheres containing variable amounts of O_2_. Such coefficients, once generated, could then be used in both 1-D and 3-D photochemical models. We are pursuing this project with the Fernández *et al*. research group as this paper is being submitted.

We note that the excellent agreement between the ozone columns in the 1-D model and ROCKE-3D could be accidental. Photolysis of O_2_ in ROCKE-3D does not cover the full SRB, or include absorption of UV radiation by CO_2_ and H_2_O, and yet the columns agree for the most part to within 3 DU. The lack of sensitivity of surface OH to the atmospheric O_2_ level in ROCKE-3D is simply because ROCKE-3D does not include photolysis of H_2_O at SR band wavelengths.

Thinking more generally, it is important to perform intercomparisons among different types of models to help us better understand the palaeoatmosphere or the atmospheres of other Earth-like planets. Such intercomparisons are done routinely with complex climate models used to study the effects of anthropogenic warming. Very little work has been done to intercompare the types of models studied in this paper. An exception is the recent study by Harman *et al*. [51] in which three different low-O_2_ photochemical models were compared to determine why some produced high abiotic O_2_ levels whereas others did not. We hope to continue and expand the model intercomparison project started here, after making sure that all models calculate O_2_ and H_2_O photolysis rates accurately.

## 7. Conclusion

In conclusion, we find that the reasons for some of the discrepancies between the WACCM6 model and previous 1-D models of ozone evolution have been resolved, while others have not. The short lifetime of methane at low pO_2_ (≤ 0.1 PAL) found by C22 [4] is an artefact of WACCM6's neglect of scattering and absorption of solar UV radiation by other gases at wavelengths within the O_2_ Schumann–Runge bands. This produced tropospheric OH concentrations that were unrealistically high. Thus, the possibility remains that methane could have been an important greenhouse gas during the Proterozoic eon. That said, the Kasting group 1-D model does not handle this problem rigorously, either. Work is underway to correct these deficiencies in both models.

The reasons for the lower ozone column depths calculated by the WACCM6 model compared with those predicted in 1-D are only partly understood. Odd oxygen production, as well as loss processes for O_3_ like destroying by NO_x_, ClO_x_, HO_x_ in different models should be carefully tested. The use of fixed mixing ratio lower boundary conditions for biogenic trace gases, especially CH_3_Cl, in WACCM6, as compared with the fixed flux boundary conditions used in the 1-D model, can contribute to some discrepancies on the ozone column depth, but needs more evidence from the other two models to determine whether this is a major factor, or not. But a major part of the discrepancy in ozone column depths could be caused by the different treatments of vertical transport in the two models, along with different methods of parametrizing upper tropospheric H_2_O concentrations. If so, the more realistic treatment of convection and transport in the 3-D models should cause those models to be preferred. Future intercomparisons between these and other ozone evolution models are planned to resolve this issue.

## Data Availability

All three models are publicly available. The source code for the 1-D photochemical model is available on Github: https://github.com/AoshuangJi/1-D-photochemical-model and has been archived within the Zenodo repository: https://doi.org/10.5281/zenodo.7818127. The specific release for WACCM6 used in this paper is CESM2.1.3, which can be downloaded from the following website: https://escomp.github.io/CESM/versions/cesm2.1/html/downloading_cesm. The ROCKE-3D model is available through: https://simplex.giss.nasa.gov/gcm/ROCKE-3D/. Data are available through the Dryad Dataset: https://doi.org/10.5061/dryad.kh18932b3 [52] and the Zenodo repository: https://doi.org/10.5281/zenodo.7821813 [53]. The data are provided in the electronic supplementary material [54].
